# Site-specific chelator-antibody conjugation for PET and SPECT imaging with radiometals

**DOI:** 10.1016/j.ddtec.2018.10.002

**Published:** 2018-10-24

**Authors:** Mauricio Morais, Michelle T. Ma

**Affiliations:** School of Biomedical Engineering and Imaging Sciences, King’s College London, St. Thomas’ Hospital, London SE1 7EH, United Kingdom

## Abstract

Antibodies and their derivatives radiolabelled with positron- and gamma-emitting radiometals enable sensitive and quantitative molecular Positron Emission Tomography (PET) and Single Photon Emission Computed Tomography (SPECT) imaging of antibody distribution in vivo. Chelators that are covalently attached to antibodies allow radiolabelling with metallic PET and SPECT radioisotopes. Conventional strategies for chelator-protein conjugation generate heterogeneous mixtures of bioconjugates that can exhibit reduced affinity for their receptor targets, and undesirable biodistribution and pharmacokinetics. Recent advances in bioconjugation technology enable site-specific modification to generate well-defined constructs with superior properties. Herein we survey existing site-specific chelator-protein conjugation methods. These include chelator attachment to cysteines/disulfide bonds or the glycan region of the antibody, enzyme-mediated chelator conjugation, and incorporation of sequences of amino acids that chelate the radiometal. Such technology will allow better use of PET and SPECT imaging in the development of antibody-based therapies.

## Graphical abstract

**Figure:**
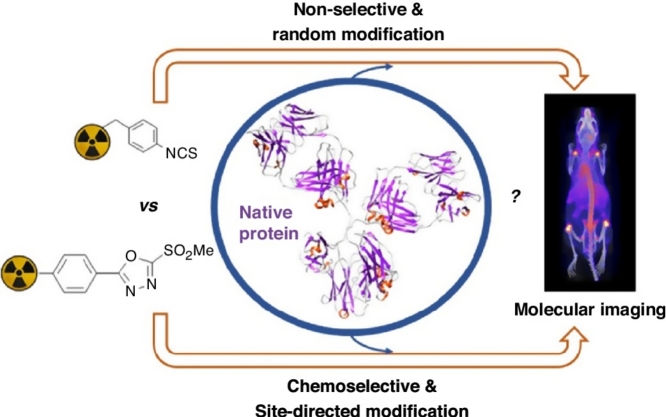

## Introduction

Monoclonal antibodies (mAbs) have demonstrated exquisite sensitivity and selectivity for their target cell surface receptors in vivo [1]. As well as being important in clinical therapies [2], [3], mAbs can be used as in vivo vectors, to deliver an additional therapeutic payload (e.g. small-molecule cytotoxic compounds [4], [5], [6] or radiotherapeutic isotopes [7], [8]) or, in combination with an imaging probe (e.g. a gamma or positron-emitting radionuclide, or an optically active molecule), to visualize the in vivo distribution of target cell surface receptors.

Antibodies labelled with a gamma- or positron-emitting radionuclide can be used to quantitatively image the biodistribution of the radiolabelled-antibody using whole body Single Photon Emission Computed Tomography (SPECT) or Positron Emission Tomography (PET) respectively. Such radiolabelled mAbs are extremely useful for both preclinical and clinical development of antibody-based therapies, enabling (i) non-invasive detection of the target receptors’ expression, including any potential heterogeneity in expression, (ii) estimation of an antibody’s biodistribution, therapeutic index and pharmacokinetics by quantification of antibody distribution in target and normal tissues, and (iii) prediction and assessment of a patient’s response to a specific mAb therapy by imaging with the radiolabelled antibody [9].

Radioactive metal ions are well-suited to radiolabelling antibodies for PET and SPECT imaging. Compared to non-metallic radionuclides, radiometals allow for simple radiolabelling procedures: typically, a chelator is firstly covalently attached to the antibody, and once conjugated, the chelator binds the radiometal. The half-lives of many of the metallic radionuclides, including zirconium-89 [9] (78 h half-life) for PET, and indium-111 [10] (67 h half-life) for SPECT, more closely match the time required for antibodies to clear circulation and accumulate in target tissue (1 day–1 week) than non-metallic radionuclides such as fluorine-18 (119 min half-life). Antibodies labelled with PET, SPECT and radiotherapeutic radioisotopes of iodine have been extensively studied both preclinically and clinically [11], however, many of these are subject to deiodination in vivo. Advances in radiochemical methodology have increased stability of radioiodine-antibody constructs [12], [13], however, this is beyond the scope of this review.

### Antibody structure

**Fig. 1:**
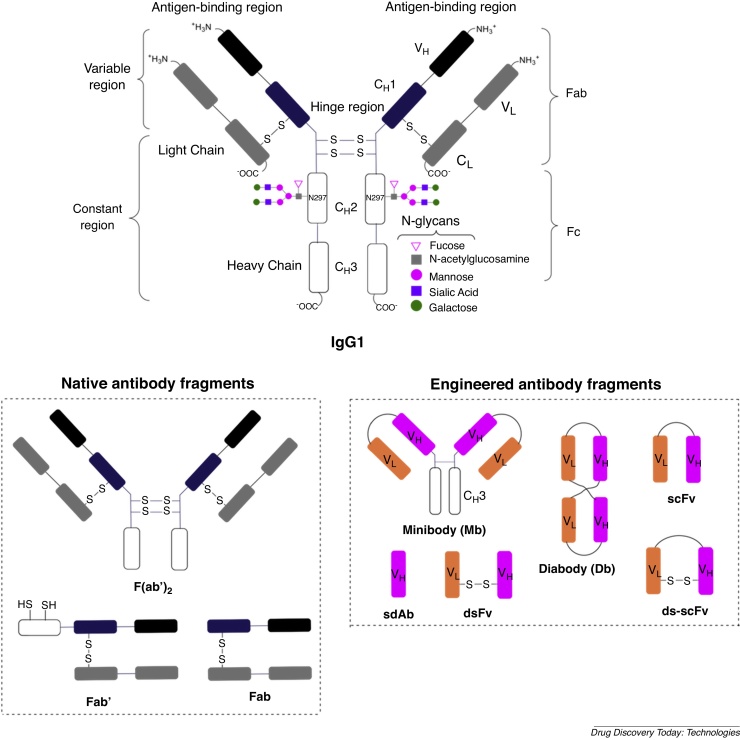
Structure of IgG1 antibodies and smaller, engineered fragment antibodies.

Immunoglobulin type 1 antibodies (Fig. 1) (IgGs) are the most commonly used type of mAb for pharmaceutical applications. They are approximately 150 kDa, and are composed of two identical polypeptide “heavy chains” paired with two “light chains”. They include a fragment antigen-binding (Fab) region, a fragment crystallisable (Fc) region, two disulfide bonds in the hinge region and a conserved glycosylated position at N297 of each heavy chain [1], [5]. Smaller derivatives of IgGs that include the targeting variable region of the Fab region have also been engineered. Although they generally exhibit lower accumulation at disease sites, they clear circulation faster than full-length IgGs [14]. Recently described radiolabelled-immunoconjugates include both full-length IgG mAbs, and smaller fragment derivatives [14], [15]. Radionuclide imaging with these smaller derivatives has shown that high “target to non-target” contrast can be achieved at early time points (1–12 h) following radiotracer administration. In contrast, full-length IgG antibodies require significantly greater time periods (1 day–1 week) to enable the antibody to accumulate at target tissue and clear circulation.

### Chelators for radiometal-antibody imaging

Metallic radioisotopes are incorporated into an antibody via a chelator. Many factors influence the choice of a metallic radioisotope, including the imaging modality (PET or SPECT/γ-scintigraphy imaging), matching of the half-life of the radioisotope to the pharmacokinetics of the vector, and the availability of the radioisotope itself. The chelator binds the radiometal, and the resulting radiometal–chelator complex will ideally possess both high thermodynamic and kinetic stability. This high stability is essential to ensure that the radiometal remains bound to the antibody in vivo. We [16], [17], [18] and others [19], [20], [21] have reviewed existing and new chelator technology for radiometal-based PET and SPECT imaging. Here, we simply include a list of commonly used imaging radiometals (Table 1) for ease of reference.

**Table 1:** Decay properties and production methods for selected radiometals used in PET and SPECT imaging.

Radiometal	Half-life	Mode of decay (%)	Production mode	Application
^99m^Tc	6.0 h	IT^a^ (100)	^99^Mo/^99m^Tc generator	SPECT
^111^In	2.83 d	EC^b^ (100)	^111^Cd(p,n)^111^In	SPECT
^67^Ga	3.27 d	EC (100)	^68^Zn(p,2n)^67^Ga	SPECT
^68^Ga	68 min	*β*^+^^c^ (90)	^68^Ge/^68^Ga generator	PET
^89^Zr	78.4 h	EC (77) *β^+^* (23)	^89^Y(p,n)^89^Zr	PET
^64^Cu	12.7 h	*β*^+^ (18) EC (43) *β*^−^d (39)	^64^Ni(p,n)^64^Cu	PET

a IT = isomeric transition.

b EC = electron capture.

c *β*^+^ = positron emission.

d *β*^−^ = beta emission.

### Attaching chelators to antibodies

In chelator-antibody conjugation reactions, the antibody and chelator contain complementary reactive functional groups for attachment to each other. There are several requirements for this covalent attachment:

(i) mild conjugation (and subsequent radiolabelling) reaction conditions are essential to preserve the tertiary and quaternary structure of the antibody;
(ii) the new covalent link between the chelator and protein must be stable under physiological conditions; and
(iii) the covalent modification must not compromise the binding affinity and specificity of the protein.

**Fig. 2:**
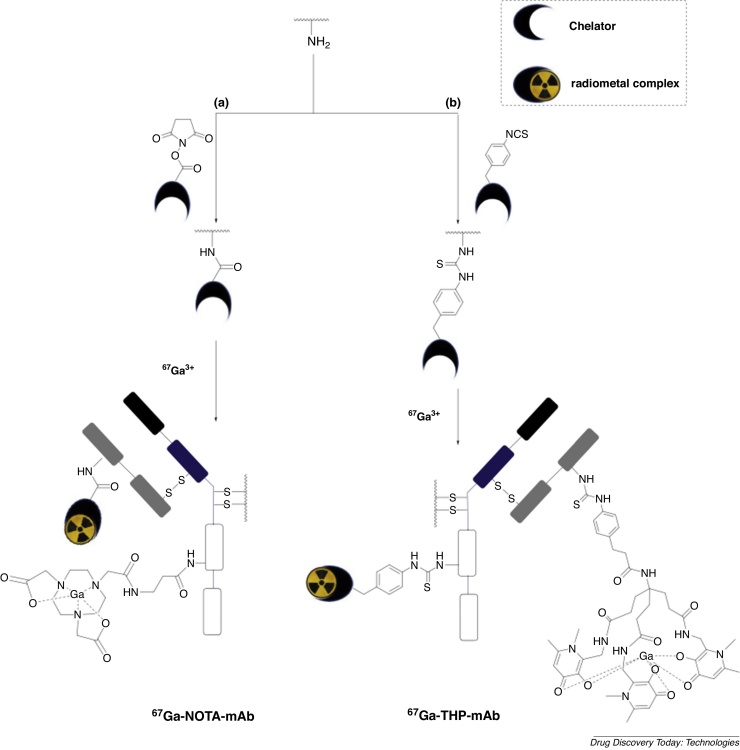
Conventional bioconjugation methods used for lysine modification in nuclear imaging: (a) *N*-hydroxysuccinimide ester and (b) isothiocyanate derivatives are commonly used. We illustrate attachment of a NOTA (1,4,7-triazacyclononane-*N*,*N*′,*N*″-triacetetate) chelator and a THP (tris(hydroxypyridinone)) chelator. Metal complex charges are excluded.

**Fig. 3:**
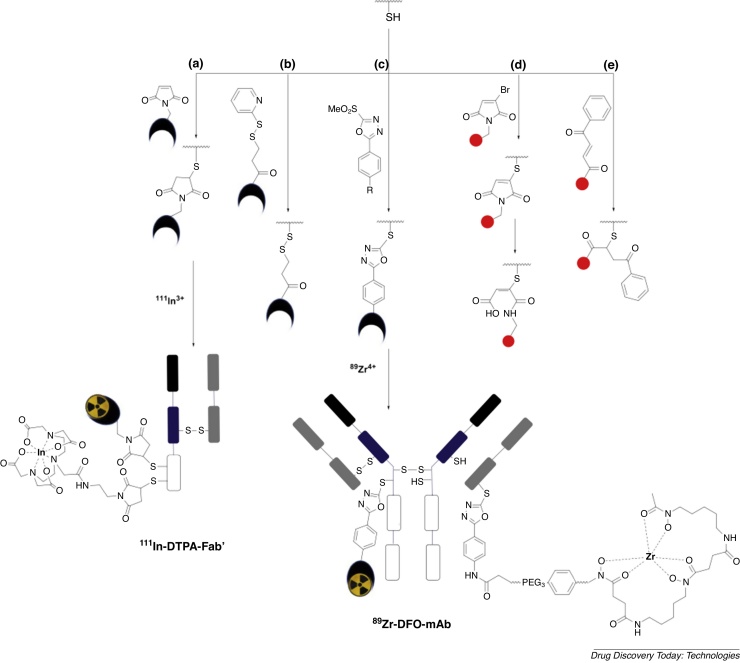
Thiol-reactive compounds including (a) maleimide, (b) pyridyldithiopropionate, (c) methylsulfonyl phenyloxadiazole, (d) monobromo malelimide and (e) carbonylacrylic derivatives of chelators and fluorophores can be used to incorporate chelators into antibodies and their derivatives. Here we also illustrate incorporation of a ^89^Zr-DFO (desferrioxamine) chelator and an ^111^In-DTPA (diethylenetriamine pentaacetate) chelator. Metal complex charges are excluded.

Most of the conventional functionalities used to attach chelators to proteins consist of reactive electrophilic groups such as isothiocyanates, *N*-hydroxysuccinimide esters (Fig. 2) and anhydrides that react with solvent accessible primary amines of lysine side chains, and maleimides that attach via Michael addition to the thiols of reduced cysteine side chains (Fig. 3a) [18], [22], [23], [24], [25], [26].

In these conventional conjugation reactions, the presence of multiple solvent-accessible amino acids in proteins leads to a lack of both stoichiometric control and site-specificity. The resulting heterogeneous mixtures of chelator-protein conjugates can exhibit suboptimal pharmacokinetics and decreased affinity for target receptors [27], [28], [29]. Additionally, the heterogeneous nature of the conjugates is potentially a barrier to regulatory approval of their clinical application and development.

Significant efforts over the last decade have resulted in new site-selective conjugation methods for attaching chelators and other cargoes (fluorescent molecules, small molecular weight drugs) to antibodies [30], [31], [32], [33], [34], [35], [36], [37], [38], [39]. Such an approach (often described as orthogonal) uses complementary pairs of functional groups that react chemoselectively with each other. It involves appending one functional group to the chelator, and the other to the protein, followed by reaction between the two motifs. Ideally, this reaction will proceed in water at near-neutral pH and ambient temperature (25–37 °C) to avoid protein denaturation or degradation.

“Click chemistry” is one such approach [40], [41]: for example, engineering cyclooctyne and azide groups into a chelator and antibody to give a triazole-containing bioconjugate, can provide this desired chemoselectively. However, this approach requires modification of the antibody prior to the conjugation reaction itself, and if the azide/octyne is incorporated via a *non-specific* lysine modification, as is often the case, site-selectivity is not actually achieved [42], [43], [44], [45]. There are elegant examples in which reactive “click” groups have been site-selectively introduced into antibodies [30], [46], and examples are included in relevant sections below.

Here, we capture an overview of site-selective conjugation methods used to prepare radiometal-labelled antibodies and antibody derivatives for PET and SPECT imaging. This includes site-directed modification of cysteines/disulfide bonds and the glycan region of the antibody, enzyme-mediated conjugation, and incorporation of sequences of amino acids that coordinate the radiometal. Others have also recently reviewed this area, including an elegant and detailed survey of site-selective antibody conjugation methods used for molecular imaging with *both* optical and radionuclide imaging labels, and site-selective methods to incorporate radiotherapeutic isotopes [47], [48].

## Cysteine modification

Cysteines are useful for selective protein modification for several reasons:

(i) cysteine has a low abundance (1–2%) in living organisms [49], [50], and so the probability of adversely affecting the pharmacokinetics of proteins by attachment of too many chelators is relatively low;
(ii) the nucleophilicity of the cysteine deprotonated thiol group (p*K*_a_ of ∼ 8.3) exceeds the reactivity of other nucleophilic groups of amino acids in proteins [51], [52];
(iii) single, solvent-exposed cysteine residues can be engineered into antibodies and their derivatives [53], [54], enabling site-selective attachment of cargo, including radiolabelled chelators. In many cases, such cysteines are introduced at the carboxyl terminus of the protein, to minimise the likelihood that the modification will impair protein structure and activity. These new cysteine residues are often “capped” by a single cysteine amino acid or thiol-containing small molecule [55], or form protein homodimers via intermolecular disulfide bonds [56]. Thus, a reduction step is required to generate a reduced thiol for subsequent conjugation. Full-length antibodies [57] and other small proteins [58], [59] have been modified using this approach.

### Conjugation via C—S bonds

The most widely employed method of conjugating antibodies via cysteines involves a Michael addition reaction of a thiol with a maleimide to form a succinimidyl thioether adduct (Fig. 3a).

Radionuclide imaging studies have demonstrated that succinimidyl thioether linkages have superior stability compared to disulfide linkages. For example, maleimide and pyridyldithiopropionate (Fig. 3a/b) groups appended to DTPA chelators have been reacted with terminal cysteine residues of an anti-carcinoembryonic Fab’ [60]. The new DTPA-Fab’ conjugates were radiolabelled with ^111^In, and their biodistribution assessed in mice bearing colorectal carcinoma tumours. The (maleimide-derived) thioether-linked radiotracer enabled efficient tumour targeting, whereas the (pyridyldithiopropionate-derived) disulfide-bridged analogue showed poor biodistribution with high kidney uptake and poor tumour targeting, due to in vivo cleavage of the S—S linkage.

Maleimide derivatives have been widely used to incorporate chelators, via cysteine thiols, into antibody derivatives and proteins [26], [55], [56], [57], [58], [59], and many chelator-maleimide reagents are commercially available. However, maleimide conjugates suffer from instability: the thioether can undergo a retro-Michael reaction, converting back to the starting thiol and maleimide. The maleimide motif, still attached to its payload, reacts with endogenous molecules containing bioavailable thiols, such as glutathione and albumin [61], [62], [63], [64]. In radionuclide imaging, this can potentially result in accumulation of radioactivity at off-target sites, decreasing image contrast, sensitivity and the ability to quantify protein biodistribution.

Several new cysteine-reactive reagents that provide enhanced conjugate stability have been developed [64], [65], [66], [67]. Following reduction of antibody disulfide bonds (typically with tris(2-carboxyethyl)phosphine hydrochloride), methylsulfonyl phenyloxadiazole (Fig. 3c) derivatives bearing DFO and DTPA chelators have been selectively attached to cysteines of trastuzumab, cetuximab and huA33 antibodies [68], [69]. These chelator-antibody conjugates contained an average of 1.4–2.2 chelators per antibody and gave ^89^Zr-DFO-mAbs and radiotherapeutic ^177^Lu-DTPA-mAbs that demonstrated higher C—S bond stability in serum than maleimide derivatives. Furthermore, methylsulfonyl phenyloxadiazole-derived ^89^Zr-DFO-huA33 antibody demonstrated superior in vivo targeting behaviour compared to its maleimide-derived analogue: the former resulted in higher tumour-to-background activity ratios in a murine model bearing huA33 antigen-expressing colorectal cancer xenografts.

Monobromo maleimide (Fig. 3d) [67], [70] and carbonylacrylic (Fig. 3e) [71] reagents have been used to generate stable protein conjugates via native, single accessible cysteines, although to date, they have not been used in radionuclide imaging. Monobromo maleimides enable substitution of the bromo group by a reactive thiol, generating a thiol-maleimide that can be hydrolysed (ring-opened) to give a C—S bond that is stable to undesirable retro-Michael deconjugation [67], [70].

### Modifying antibody disulfide bridges

Single cysteine-targeted conjugation strategies are well-suited for site-specific modification of Fab or scFv fragments that contain single, exposed cysteine residues, however, they are not ideal for generating well-defined conjugates of IgG antibodies. IgG proteins contain four solvent-accessible interchain disulfide bridges in the protein hinge region, and their reduction creates eight reactive thiols. Conjugation to these reduced species results in heterogeneous mixtures of conjugates, and the cleavage and modification of these disulfide bridges (with up to eight copies of cargo) can lead to adverse pharmacokinetics, and reduce the metabolic stability of IgG antibodies in plasma [72], [73]. Additionally, reduced thiol groups that do not participate in bioconjugate reactions can undergo oxidative intramolecular reactions with other thiols, often resulting in disulfide scrambling that disrupts the structure and function of the protein.

**Fig. 4:**
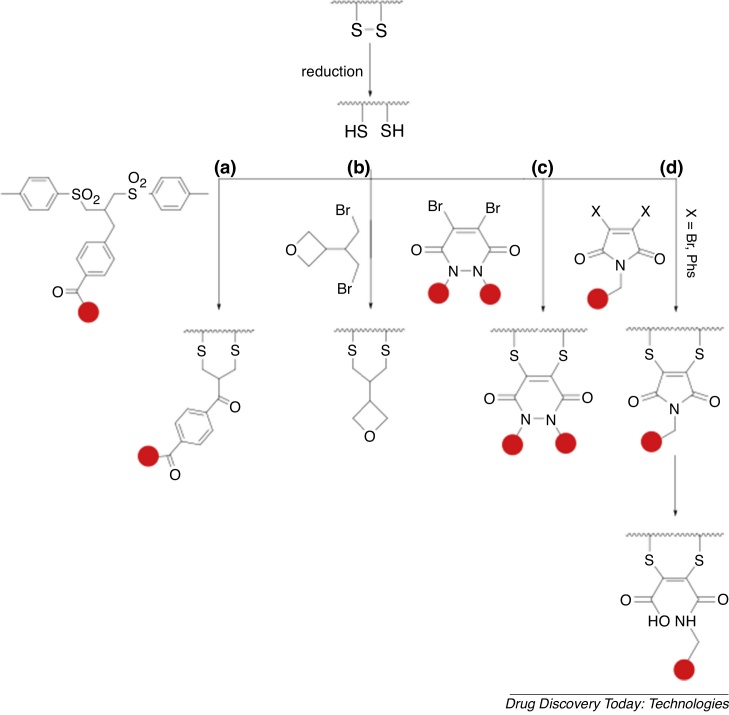
New conjugation technology that enables disulfide rebridging includes derivatives based on (a) bissulfone, (b) dibromoalkyl oxetane, (c) dibromopyridazinediones and (d) dibromo/dithiophenyl maleimides. This technology has not been applied to radionuclide-labelling of antibodies, but is a promising future avenue.

Recent research has produced functional species that react with two reduced thiol groups of antibodies, thus enabling concomitant attachment of functional groups (for example, fluorophores) *and* re-bridging of two cysteines. It is important that the re-bridging motif reacts rapidly with both disulfide-derived reduced thiols, to avoid incorrect re-bridging, and thus preserve structure and function of the protein [74], [75], [76], and that the resulting re-bridged covalent bonds are unreactive towards serum thiols. Novel thiol-stable chemical technologies have been successfully applied to modification of disulfide bonds in mAbs and their derivatives. These include bissulfone derivatives (Fig. 4a) [77], [78], [79], [80], dibromoalkyl oxetane derivatives [81] (Fig. 4b), trivalent arsenous acid [82], dibromopyridazinediones (Fig. 4c) [73], [83], and disubstituted maleimides (Fig. 4d) [84], [85], [86], [87], [88]. Bridged bisthiomaleimide (derived from disubstituted maleimides) can be hydrolysed to dithiomaleamic acid under mildly basic conditions, generating homogenous antibody conjugates that are unreactive towards serum thiols and do not undergo retro-Michael reactions in biological media, unlike conventional maleimide derivatives [85], [89], [90]. These technologies have not been applied to radionuclide imaging, but could be advantageous for future antibody-based radionuclide imaging.

## Glycan modification

IgG proteins contain two conserved post-translational modification glycosylation sites (Fig. 1) that can be chemically modified to enable site-selective attachment of chelators. This conjugation strategy is appealing because:

(i) modification at these sites will not compromise antigen binding properties as they are distal to the Fab region;
(ii) there are two attachment sites available per antibody; and
(iii) there are several chemoselective/orthogonal reactions enabling modification of glycans and hexose groups.

This method is not suitable for smaller antibody fragments that lack glycans, or for IgG1 antibodies whose function requires the presence of the native (unmodified) Fc regions for binding to Fc-receptors (such as those involved in immune responses).

### Importance of glycan modification chemistry to clinical antibody imaging

Glycan-based modification chemistry has been critically important in the clinical development of molecular imaging with antibodies in nuclear medicine [91]. The first FDA-approved imaging radioimmunoconjugate specifically incorporated ^111^In into the satumomab antibody via a DTPA chelator at the glycan region (see below). ^111^In-DTPA-satumomab targets a tumour-associated glycoprotein, TAG-72, expressed in several cancers, including colorectal and ovarian cancers [92]. Clinical γ-scintigraphy and SPECT imaging trials in over 1000 patients have demonstrated ^111^In-DTPA-satumomab’s utility in detecting colorectal and ovarian cancer lesions, and in combination with other diagnostic tests, informing clinical decision-making, including treatment and surgery.

Another FDA-approved antibody imaging agent, ^111^In-capromab pendetide (also known as Prostascint), similarly incorporates a DTPA chelator using this glycan technology [91]. ^111^In-capromab pendetide targets the prostate specific membrane antigen (PSMA) expressed in prostate cancer, and has demonstrated potential in assisting in (i) the staging of prostate cancer, particularly in identifying soft tissue metastases, and (ii) locating prostate cancer tumours when diagnostic blood tests indicate disease recurrence [93]. Although ^111^In-capromab pendetide has not demonstrated high positive predictive value and specificity for clinical management of prostate cancer, it has been fundamentally important in the development of radionuclide molecular PET and SPECT imaging of its target, PSMA. Several PSMA-targeted PET and SPECT imaging agents are currently being clinically developed [94], [95], [96], [97], after showing high diagnostic utility in prostate cancer management.

### Reaction with oxidised hexose groups

**Fig. 5:**
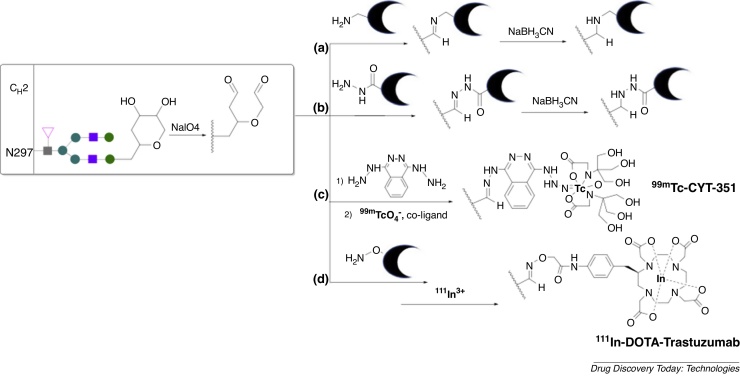
Oxidation of antibody glycan sites generates reactive aldehydes that selectively react with: (a) amines, (b/c) hydrazines and (d) hydroxylamines enabling site-specific attachment of chelators. Such methods have been used to incorporate a ^99m^Tc-tricine radiolabel and an ^111^In-DOTA (1,4,7,10-tetraazacyclododecane-1,4,7,10-tetraacetate) radiolabel. Metal complex charges are excluded.

One of the most widely used methods for site-specific modification of glycan sites relies on the generation of an aldehyde group by oxidising the *cis*-glycol groups of terminal hexoses (Fig. 5a–c), commonly with sodium periodate [33], [98], [99], [100].

The generated aldehydes can be reacted with chelator derivatives containing pendant primary amines, generating an imine conjugate. The resulting imine conjugate can be further modified by reduction with cyanoborohydride to form an amine, preventing in vivo hydrolysis of the newly generated linker (Fig. 5a) [101]. ^111^In-DTPA-satumomab and ^111^In-capromab pendetide both incorporate DTPA chelators via this strategy [91]. Compared to antibodies in which the radiolabel is incorporated via solvent-accessible lysines, radiolabelling via site-specific glycan modification leads to increased radioactivity accumulation in tumours and decreased off-target tissue radioactivity concentration [91], [102].

Aldehydes of modified glycans can also be reacted with hydrazides (Fig. 5b), and in some cases the resulting hydrazone bioconjugates have been reduced to generate more stable chelator-antibody derivatives [103], [104], [105], [106]. A prostate-targeting hydrazide-linked chelator-antibody (CYT-351) labelled with ^99m^Tc (Fig. 5c) has enabled planar and SPECT imaging of prostate cancer in patients [103].

Glycan-derived aldehydes and ketones have been reacted with *O*-alkyl hydroxylamines to form oxime ethers (Fig. 5d). This covalent attachment is more stable than imine or hydrazone linkers and does not require subsequent reduction steps [107]. This method has been used to prepare a ^111^In-DOTA-trastuzumab species, which demonstrates conserved immunoreactivity for target HER2 receptors both in vitro and in tumour bearing mice [108].

### Enzymatic modification of glycans

**Fig. 6:**
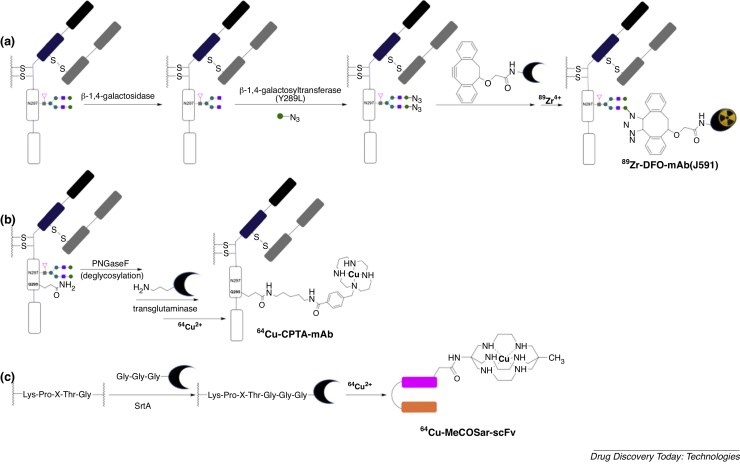
Site-directed enzyme-mediated bioconjugation methods utilise (a) β-1,4-galactosyltransferase (Y289L) (b) transglutaminase and (c) sortase A (SrtA) enzymes. These have been used to incorporate ^89^Zr-labelled DFO chelator and ^64^Cu-labelled chelators, CPTA (a 1,4,8,11-tetraazacyclotetradecane derivative) and MeCOSar (a 3,6,10,13,16,19-hexaazabicyclo[6.6.6]icosane derivative). Metal complex charges are excluded.

Although full-length antibodies have been successfully modified via glycan oxidation/conjugation, in some cases, the harsh oxidation conditions can lead to inadvertent oxidation of methionine residues of the antibody, reducing the serum half-life of the antibody [109]. A dual enzyme approach that enables functionalisation of glycans can avoid this: the enzyme β-1,4-galactosidase removes the terminal galactose residues of antibodies, and following this, the mutant enzyme β-1,4-galactosyltransferase (Y289L) incorporates a modified galactose unit containing a reactive functional group at this site [30], [110]. This technology has been applied to site-directed radiolabelling of a J591 prostate-cancer targeting IgG [30], where the terminal galactose residue was substituted for a galactose motif containing an azide. In a copper-free azide/dibenzocyclooctyne cycloaddition “click” reaction, the DFO chelator was then conjugated to the antibody and radiolabelled to give ^89^Zr-DFO-J591 IgG (Fig. 6a). This derivative showed higher tumour uptake than the randomly modified analogue (via lysine modification with isothiocyanate). Using this strategy, dual fluorescent, ^89^Zr-radiolabelled antibodies were prepared and showed efficacy in imaging A33 transmembrane glycoprotein expression in a colorectal tumour mouse model.

## Enzyme-mediated conjugation

Recently, protein technology has been developed to enable enzyme-mediated, site-specific conjugation of a cargo to target antibodies and proteins [111], [112], [113], [114]. These methods make use of an enzyme that recognises two complementary motifs on the targeting antibody/protein and the cargo-containing compound. Using these two complementary motifs, the enzyme catalyses covalent attachment of the targeting antibody/protein to the cargo. The use of β-1,4-galactosyltransferase (Y289L) with DFO chelator (Section “Reaction with oxidised hexose groups”) is an example of this. Other examples of enzyme-mediated, site-specific conjugation of chelators to proteins also exist. Like reactions involving β-1,4-galactosyltransferase (Y289L), such reactions achieve chemoselective chelator conjugation under mild conditions that do not denature the antibody.

### Transglutaminase

The bacterial transglutaminase (BTG) enzyme catalyses the formation of a new amide bond between the primary amine of a lysine side chain and the γ-carboxamide group of a glutamine side chain. Many 5-aminopentyl derivatives can act as lysine side chain surrogates and are recognized by BTG, however, BTG is only reactive towards glutamine side chains located in flexible regions of proteins/biomolecules. Glutamine side chains of IgG antibodies are thus normally unreactive to BTG. Removal of glycans from position N297 of an IgG antibody, using the enzyme *N*-glycosidase F (PNGaseF), results in increased flexibility of this antibody region. This gives rise to the increased BTG-catalysed reactivity of a glutamine residue, Q295, in close proximity to N297, and chelators bearing 5-aminopentyl groups can be site-selectively introduced into this position in the presence of BTG [115], [116]. Reaction of either anti-L1-CAM chCE7 antibody or rituximab antibody (Fig. 6b), firstly with PNGaseF to remove N297 glycans, and secondly, with bifunctional chelators attached to 5-aminopentyl groups in the presence of BTG, results in IgG mAbs bearing only two chelators per antibody, attached at position Q295 [115]. Radionuclide imaging and biodistribution studies in tumour-bearing mice showed that this site-specific conjugation strategy led to radiolabelled antibodies that provided higher tumour to non-target organ contrast, compared to antibodies radiolabelled using conventional, non-specific methods (lysine modification).

### Sortase A

Bacterial enzyme sortase A (**SrtA**) catalyses a transpeptidase reaction between an N-terminal glycine, and a specific amino acid sequence, -Lys-Pro-X-Thr-Gly- (where X is any amino acid) [117], [118], [119]. SrtA (i) recognizes and cleaves this peptide sequence between threonine and glycine, and (ii) catalyses the formation of a new amide bond between threonine and an N-terminal glycine-containing species. Using this protein chemistry, a macrobicyclic sarcophagine (a 3,6,10,13,16,19-hexaazabicyclo[6.6.6]icosane derivative) that binds ^64^Cu has been site-selectively appended to a scFv that targets GPIIb/IIIa glycoprotein receptors overexpressed in thrombosis, atherosclerosis and inflammation. Two different conjugation strategies have been described. In the first SrtA-catalysed conjugation, the scFv, engineered to contain a -Lys-Pro-X-Thr-Gly- sequence at the C-terminus, was reacted with an N-terminal glycine residue attached to a sarcophagine chelator (Fig. 6c) [119]. In the second, the same scFv (in the presence of Srt A) was reacted with an N-terminal glycine attached to a strained cyclooctyne [118]. Once purified, the new scFv-cyclooctyne bioconjugate was reacted chemoselectively with an azide appended to a sarcophagine ligand. Whilst the first approach required fewer derivatisations to incorporate a chelator, the second approach yielded a scFv-octyne bioconjugate that is more versatile — it can site-selectively incorporate any label bearing an azide motif. Both ^64^Cu-sarcophagine labelled scFv conjugates enabled PET imaging of GPIIb/IIIa glycoprotein receptors expressed in thrombosis.

## Amino acid coordinating sequences

Peptides and proteins can bind to metal ions via amino acid side chains, carboxylate groups of C-termini, amine groups of N-termini, and N atoms of amide groups of the peptide backbone. Particular sequences of amino acids, containing several metal-binding ligands, enable direct radiometal complexation by the protein, without the requirement of a synthetic chelator. In such cases, the radiometal-binding amino acid sequence is simply engineered into the protein at the desired location.

### His tags

The hexahistidine sequence, or His_6_ tag, originally developed to aid in protein purification, has been prevalently applied to incorporate the SPECT isotope, ^99m^Tc (in the form of ^99m^Tc(CO)_3_^+^), into antibody derivatives [120], [121], [122], [123], [124], [125], [126]. The His_6_ tag is commonly incorporated at the C-terminus of targeting proteins, although N-terminal incorporation is also possible. Many ^99m^Tc-labelled proteins have been radiolabelled using this strategy, with recent examples including a HER2-targeted sdAb [123], a α_v_β_6_ integrin-targeted diabody [121] and a PSMA-targeted diabody [122]. SPECT imaging with these radiotracers enables visualisation of target tumour tissue. Computational modelling suggests ^99m^Tc(CO)_3_^+^ coordinates via two imidazole groups of a His_6_ tag [127]. Modifications to the His_6_ tag have improved protein radiolabelling and biodistribution. For example, the inclusion of a thiol-containing cysteine residue to a His_6_-containing sequence can increase metallolabelling efficiency [128]. Alternatively, substitution of His residues in the His_6_ amino acid sequence can increase hydrophilicity and negative charge of a radiolabelled protein, which in turn, can decrease radioactivity retention in non-target tissue in vivo, leading to improved image contrast. For example, substituting the sequence HHHHHH for HEHEHE does not adversely affect ^99m^Tc-radiolabelling of a HER2-targeted protein, but it does decrease radioactivity retention in non-target tissue in vivo [129].

### Sequences incorporating amide-binding motifs

Several low molecular weight ^99m^Tc radiotracers, including ^99m^Tc-labelled compounds used in renal imaging and ^99m^Tc-labelled peptides that target cell surface receptors [19], [96], [130], [131], make use of deprotonated amide groups of a peptide backbone in combination with deprotonated thiols to coordinate ^99m^Tc^V^. Peptide sequences incorporating these features can be engineered into proteins for efficient binding of the ^99m^Tc^V^O^3+^ motif. The first example of this demonstrated that Gly_4_Cys, engineered into the C terminus of a scFv protein, could be applied to SPECT imaging of a scFv disease target [132], [133], [134], [135], [136]. Subsequent studies have demonstrated that a scFv protein incorporating a C-terminal ^99m^Tc^V^(O)-Gly_3_Cys sequence has more favourable biodistribution properties (faster clearance and lower off-target organ retention) than a C-terminal ^99m^Tc^I^(CO)_3_-His_6_ homologue [136]. Glycine residues can be substituted for other amino acid residues without compromising ^99m^Tc^V^(O) binding abilities [96], [134], [135], [137], however incorporation of the amino acid binding sequence Cys-Gly_x_ directly at the N-terminus (instead of Gly_x_-Cys directly at the C-terminus) can lead to release of ^99m^Tc radionuclide in vivo, compromising imaging ability [133], [134].

## Summary and concluding remarks

Advances in conjugation chemistry and protein engineering have enabled development of homogenous radiolabelled antibodies. New site-specific antibody modification strategies that have not yet been applied to PET/SPECT imaging with radiolabelled antibodies could similarly be adapted to generate well-defined chelator-antibody constructs. Several preclinical studies have highlighted that site-specifically modified antibodies have improved in vivo behaviour (higher affinity for target receptors, lower off target accumulation/persistence, better conjugate stability) relative to those modified using conventional, less specific methods. Site-specifically radiolabelled antibody-based radiopharmaceuticals will deliver new clinically-useful contrast agents for molecular PET/SPECT imaging, by (i) providing clinicians with better molecular imaging tools to predict whether a patient will respond to a particular treatment or intervention, and (ii) providing scientists with reliable tools to quantitatively map the in vivo behaviour of new antibody-based therapies and/or newly-discovered receptors that are drug targets. Such technology will also be critically important in providing antibody conjugates with precisely defined structures and stoichiometries that are acceptable to regulatory authorities.
